# Digital Atlasing and Standardization in the Mouse Brain

**DOI:** 10.1371/journal.pcbi.1001065

**Published:** 2011-02-03

**Authors:** Michael Hawrylycz, Richard A. Baldock, Albert Burger, Tsutomu Hashikawa, G. Allan Johnson, Maryann Martone, Lydia Ng, Chris Lau, Stephen D. Larsen, Jonathan Nissanov, Luis Puelles, Seth Ruffins, Fons Verbeek, Ilya Zaslavsky, Jyl Boline

**Affiliations:** 1 Allen Institute for Brain Science, Seattle, Washington, United States of America; 2 MRC Human Genetics Unit, Institute of Genetics and Molecular Medicine, Edinburgh, United Kingdom; 3 MRC Human Genetics Unit, Edinburgh and Heriot-Watt University, Edinburgh, United Kingdom; 4 RIKEN Brain Science Institute, Saitama, Japan; 5 Duke University, Center for In Vivo Microscopy, Durham, North Carolina, United States of America; 6 National Center for Microscopy and Imaging Research (NCMIR), University of California, San Diego, La Jolla, California, United States of America; 7 Department of Basic Sciences, Touro University Nevada, College of Osteopathic Medicine, Henderson, Nevada, United States of America; 8 CIBER en Enfermedades Raras 736 and Faculty of Medicine, University of Murcia, Murcia, Spain; 9 Laboratory of Neuro Imaging (LONI), University of California, Los Angeles, California, United States of America; 10 Leiden Institute of Advanced Computer Science, Leiden University, Leiden, The Netherlands; 11 San Diego Supercomputer Center, University of California San Diego, San Diego, California, United States of America; 12 Informed Minds, Wilton Manors, Florida, United States of America; Université Paris Descartes, Centre National de la Recherche Scientifique, France

Digital brain atlases are used in neuroscience to characterize the spatial organization of neuronal structures [1]–[3], for planning and guidance during neurosurgery [4], [5], and as a reference for interpreting other modalities such as gene expression or proteomic data [6]–[9]. The field of digital atlasing is extensive, and includes high quality brain atlases of the mouse [10], rat [11], rhesus macaque [12], human [13], [14], and several other model organisms. In addition to atlases based on histology, [11], [15], [16], magnetic resonance imaging [10], [17], and positron emission tomography [11], modern digital atlases often use probabilistic and multimodal techniques [18], [19], as well as sophisticated visualization software [20], [21].

Whether atlases involve detailed visualization of structures of a single or small group of specimens [6], [22], [23] or averages over larger populations [18], [24], much of the work in developing digital brain atlases is from the perspective of the user of a single resource. This is often due largely to the challenges of data generation, maintenance, and resources management [25], [26]. A more recent goal of many neuroscientists is to connect multiple and diverse resources to work in a collaborative manner using an atlas based framework [2], [19]. This vision is appealing as, ideally, researchers would be able to share their data and analyses with others, regardless of where they or the data are located. An important step in this direction is the specification of a common frame of reference across specimens and resources (either as coordinate, ontology, or region of interest) that is adopted by the community. In this perspective, we propose a collaborative digital atlasing framework for coordinating mouse brain research that allows access to data, tools, and analyses from multiple sources.

## The INCF Digital Atlasing Project

**Figure 1:**
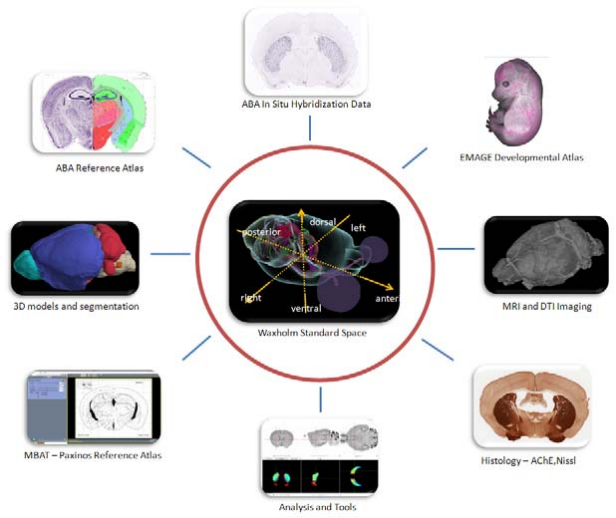
Standardizing digital atlasing. The Waxholm Space (WHS) atlas acts as the hub of an infrastructure connecting data and key reference spaces. Reference atlases that have been mapped to this space are “standardized” and can be used to share their associated data and services in a manner that is meaningful to external users. Clockwise from upper left, resources may include neuroanatomic reference atlases, large-scale gene expression databases, developmental databases, MRI and DTI imaging, histological data, analysis tools, online applications, and other 3-D anatomic models.

The International Neuroinformatics Coordinating Facility (INCF) Digital Atlasing Project (http://incf.org/core/programs/atlasing) is an international effort to design and create an atlas-based data sharing framework for the rodent brain, with initial focus on the C57Bl/6J mouse. An overview [27] and an in-depth review [28] of the goals of the project can be found at the link above. In summary, the approach is to create a canonical atlas space that is intended to encourage interoperability between existing and future mouse data resources (Figure 1). The components of this approach include 1) a standardized spatial coordinate system, 2) high resolution archival MRI and matched histological (Nissl) series data for aligning (or registering) new data to these coordinates, and 3) supporting infrastructure for data access and exchange. Through this effort a new standard is encouraged that can translate between diverse and remote atlases, similar to the Talairach and Tournoux atlas for the human brain [29]. Reference atlases (new or existing) may be registered to this standard space, and once this transformation is accessible over the Web, the atlas and related data become more useful to the outside world. Many of the resources created through this effort can be used for other strains, developmental stages, and potentially, other species.

## A Framework for Digital Atlasing in the Mouse Brain

The male adult C57Bl/6J mouse brain was chosen as the initial rodent model for its importance in genetic studies and the wide availability of data and supporting atlases. To begin, we construct a conceptual and physical atlas space called Waxholm Space (WHS, after Waxholm, Sweden, the site of the first INCF Digital Atlasing Working Group, September 2008). The coordinate system for WHS is defined as a continuous Cartesian system with the origin in the brain determined by

- the anterior commissure (AC) at the intersection between the mid-sagittal plane,
- a coronal plane passing midway (rostro-caudal) through the anterior and posterior branches of AC, and
- a horizontal plane passing midway through the most dorsal and ventral aspect of the AC.

This choice is reasonable as it is central, easily identifiable in most images, and a line passing through the center of the anterior and posterior commissures is topologically comparable at most developmental stages, except the youngest embryos.

**Figure 2:**
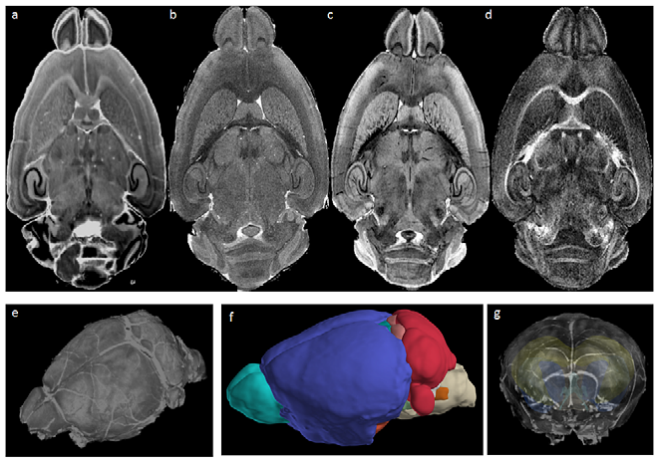
The Waxholm Space. Isotropic 21.5-µm^3^ resolution MR volumes were generated along with a complete 21-µm Nissl series of the same brain. The WHS origin is defined by the junction of the rostral and dorsal tangential planes of the anterior commissure with the mid-sagittal plane. (a) Nissl histology, (b) T1 image, (c) T2*, this image is used to provide the Nissl-MRI registration (d) fractional anisotropy image, essentially showing white matter. Several cytoarchitectonic domains can be identified at this resolution, including individual cortical layers within allocortex and isocortex, caudate putamen, and the hippocampal commisure. (e) volumetric rendering of T2* image, (f) smoothed rendering of 37 manually delimited structures, (g) 3-D rendering of WHS with gene expression correlation projection from hippocampus. Image credits: (a–d) Center for In Vivo Microscopy, Box 3302, Duke University Medical Center, Durham, North Carolina, United States of America; (e–g) Allen Institute for Brain Science, Seattle, Washington, United States of America.

Substantial new reference datasets were generated to create a canonical registration target for WHS. To capture a default overall brain geometry, magnetic resonance imaging (MRI) at microscopic resolution provides a consistent, undistorted 3-D reference frame to which histology and other data types can be mapped. WHS was formed from a single specimen, using three different MR microscopic volumes and a Nissl volume (Figure 2). While a total of over 55 3-D datasets were scanned and are available for download at http://www.civm.duhs.duke.edu/WHS/, a single canonical set was chosen as representative of the high-resolution protocol to provide the target volume for the WHS coordinate system. For this set, MR data were acquired at 9.4T in a specimen perfused with formalin/Prohance [17] and imaged with T1, T2, and T2* weighted sequences at 21.5 µm^3^ resolution with the brains in the skull. To provide a basic structural reference framework, a label volume was created that includes the delineation of 37 structures that were automatically labeled, then checked and cleaned manually using the three different MR image sets (Amira software v5.2.1, Mercury Computer Systems, Inc., Chelmsford, MA) in all three coordinate planes to ensure continuity and smoothness of the structures. We are in the process of working with the INCF Program on Ontologies of Neural Structures (PONS) group (http://www.incf.org/core/programs/pons) to create new delineations and a structure hierarchy that may be useful for mapping across different parcellation schemes and species.

To obtain a matched histological data set, three of the previous brains were frozen and cryosectioned using a low-distortion tape collection protocol [30]. Each 20-µm section was collected and Nissl stained, and following 3-D reconstruction of the Nissl slices, the Nissl volume was aligned to the T2* MR volume. By registering the Nissl volume to this space, we provide five different potential registration entry points into WHS: three MR sets with different contrast, Nissl, and 3-D structures. As other data become available (e.g., DTI, vascular, etc.) they will be registered to WHS, adding to the rich integrated environment for data sharing. The WHS datasets [31] are available in the NIfTI-1 format at the INCF software site (http://software.incf.org/software/waxholm-space/home).

**Figure 3:**
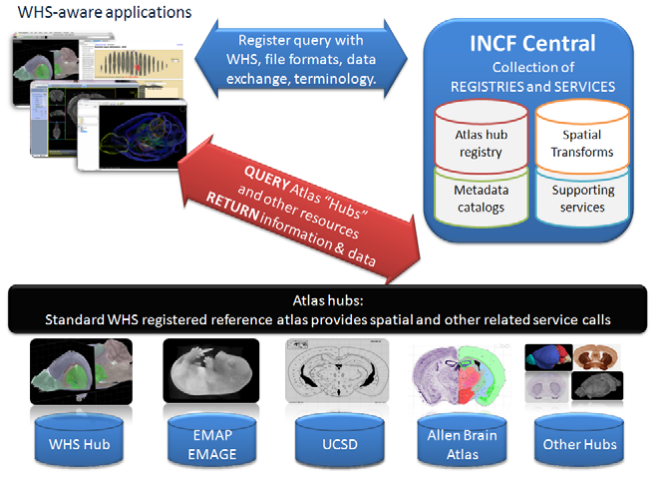
The INCF-DAI infrastructure. Individual scientific software applications will register with INCF Central for identification of file formats, transformations, standard query formats, and other essential metadata. After becoming WHS aware, key atlas hubs provide an entry point and connectivity into the community of atlases and available data resources. Presently, the Edinburgh Mouse Atlas Project (EMAP), the Whole Brain Catalog (UCSD), and the Allen Brain Atlas (ABA) have each been registered with the WHS infrastructure and can therefore initiate queries interchangeably. Other hubs can be similarly integrated and interest and resources permit.

A reference dataset is only one component of a complete digital atlasing framework. An effective system should be able to link data from multiple and remote sources for upload, analysis, processing, and sharing. The vision of the INCF Digital Atlasing Infrastructure (INCF-DAI) (Figure 3) is a collection of distributed services that support the publication, discovery, and invocation of heterogeneous atlases and resources. At the center of this architecture is INCF Central, which contains the necessary spatial and semantic definitions, central servers, and registries. This provides the means to communicate with key atlas hubs that provide an entry point for WHS registered and aware applications. In this way, any scientist's software that adheres to these standards and services could access the atlas hubs. INCF-DAI central registries keep track of the capabilities of the remote and independently supported atlas hubs, the translation information needed to map between them, and host or mirror some of the data or infrastructure when necessary.

We have recently developed an INCF-DAI prototype for the mouse brain that supports mapping between WHS reference space and the following:

- Allen Brain Atlas reference atlas (ABA, http://www.brain-map.org/) and online tools
- Edinburgh Mouse Atlas Project (EMAP, http://genex.hgu.mrc.ac.uk/)
- Whole Brain Catalog (WBC, http://wholebraincatalog.org/), including the digital Paxinos and Franklin Mouse Brain Atlas [32], hosted by the University of California, San Diego (UCSD).

This prototype presently enables lookup by anatomic structure, image retrieval, gene information retrieval, and several other atlas-specific operations. While this work demonstrates several key operations of the INCF-DAI, a full implementation of a digital atlasing system will require the support and active participation of the larger scientific community. Standards for data collection, image preprocessing, and registration transformations should also be encouraged for users to facilitate and manage data contribution. Standardization in terminology and ontology is a continuing challenge in neuroscience, and programs such as the INCF PONS (http://www.incf.org/core/programs/pons) and the Neuroscience Information Framework (NIF, http://nif.nih.gov/) are actively pursuing these goals. Ideally, these standards will be developed and shared in a manner similar to that used by the World Wide Web Consortium (W3C, http://www.w3c.org/).

## Connecting Community Resources

To illustrate the potential of the INCF Digital Atlasing framework, we integrated three major community resources into this developing infrastructure as atlas hubs: the ABA and associated tools such as the Anatomic Gene Expression Atlas (AGEA), EMAP/EMAGE for developmental mouse brain data, and the WBC, which integrates the UCSD/BIRN Smart Atlas (Spatial Mark-Up and Rendering Tool) and the Cell Centered Database (CCDB, http://www.ccdb.ucsd.edu/), including the Paxinos and Watson mouse brain atlas. Each of these atlases represents an important community resource in the rodent brain research community. To make these atlases interoperable, we registered the atlases to WHS and made their data accessible via the standards and Web services indicated above.

### The Allen Brain Atlas

**Figure 4:**
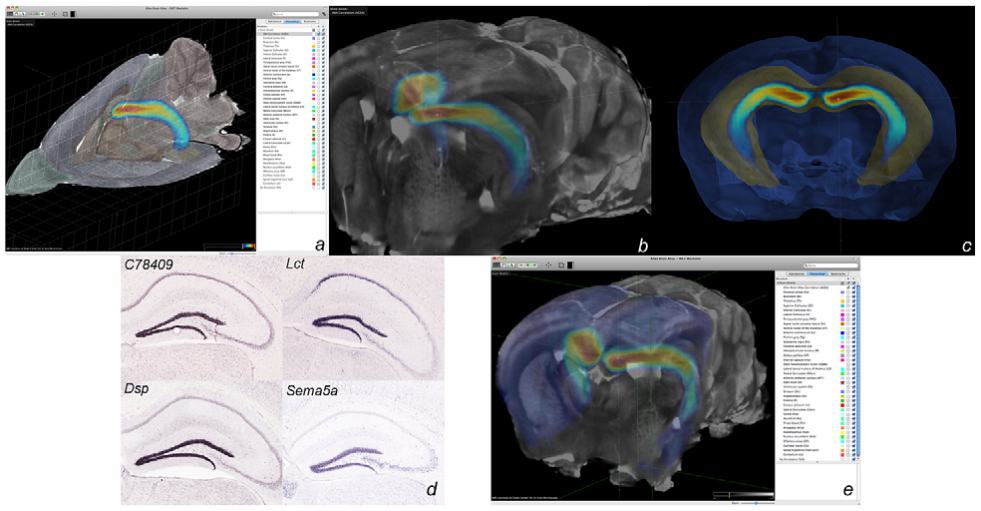
The Allen Brain Atlas and Waxholm Space. (a) WHS T1 MR image sliced on orthogonal planes. Color overlay on the planes represents segmentation of WHS anatomic regions. Blue-orange-yellow overlay is an ABA gene expression correlation map (from Anatomic Gene Expression Atlas [AGEA] online application, http://mouse.brain-map.org/agea) rendered by maximum intensity projection and showing voxels where gene expression is highly correlated with the selected point of interest (POI). This POI, in the dentate gyrus of the hippocampus, was chosen in WHS, the coordinates transformed to ABA space, the corresponding correlation volume requested from the ABA Web service. The returned volume was finally transformed back to WHS for visualization. (b) Correlation volume in (a) merged with a volume rendering of WHS cropped around the hippocampus. (c) Surface representations of hippocampus in yellow and cortex in blue show the AGEA gene expression-defined dentate gyrus in relation to an MR-defined hippocampus. (d) Top four highest correlated genes from the ABA corresponding to (c). (e) A higher resolution view of the same query within the Allen Institute Brain Explorer interface.

The ABA (http://mouse.brain-map.org/) is a database of over 20,000 in situ gene expression patterns in the adult C56BL/6J mouse brain mapped into a common coordinate system [7]. The ABA reference volume (reconstructed from Nissl-stained histological images at 25 µm^3^resolution) was registered to the WHS MRI label volume by maximizing the mutual information of manually annotated brain regions in each 3-D space where the deformation was parameterized with a multi-scale 3-D (B-spline) grid. Once the transform between ABA and WHS was established, spatial query capabilities, such as anatomic structure label and gene expression information, in WHS coordinates were implemented by transforming the point of interest from WHS to ABA space and calling existing ABA Web services. The ABA's 3-D desktop visualization application, Brain Explorer [20], was adapted to transform data between Waxholm and ABA space (Figure 4). Brain Explorer can be used to visualize gene expression patterns and correlations in these patterns between anatomic regions (http://mouse.brain-map.org/agea, [33]) using any WHS MRI dataset to query the ABA online database at regions of interest.

### The Edinburgh Mouse Atlas Project

**Figure 5:**
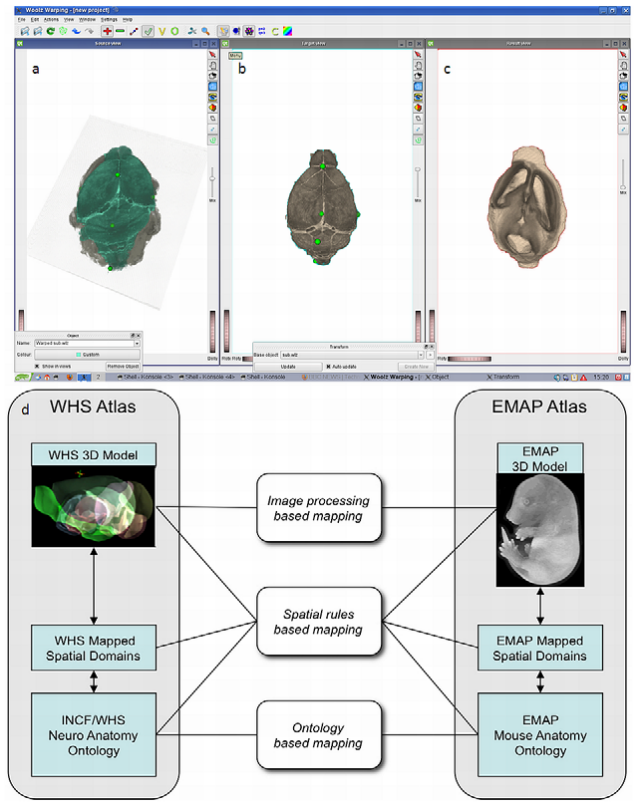
The Edinburgh Mouse Atlas Project. To link EMAP with WHS, the Waxholm volume was transformed into EMAP's native representation (b) and then mapped into EMAP Theiler Stage 23 (TS23) space, the result shown in (a). Similarly, the result of mapping the EMAP model into WHS is shown in (c). A few registration landmarks are shown in (a, b) to illustrate the process. The final transformation was established by anatomists who aligned recognizable tissue boundaries to within about five voxels in WWHS (∼100 microns). A prototype for an Edinburgh INCF hub allows access to EMAP and the related EMAGE gene expression databases available through the INCF-DAI (d). In addition to image processing–based mappings, alternative methods of mappings are being explored, including ontology-based mappings, and mapping of areas of interest across atlases using spatial rules (d).

The EMAP (http://genex.hgu.mrc.ac.uk/) is a digital atlas of mouse development associated with the EMAGE database, a resource for spatially mapped data such as in situ gene expression and cell lineage [34]. After converting WHS volumes into the EMAP native representation, the Woolz Warping Tool (http://genex.hgu.mrc.ac.uk/) was used to map WHS to EMAP space (for Thieler development stage T23) using a non-linear transformation based on a series of expert-placed landmarks (Figure 5a–b). A prototype for an Edinburgh INCF atlas hub was developed that gives access to EMAP and the related EMAGE gene expression databases available via INCF-DAI. Mapping from the adult mouse brain (WHS) to the EMAP mouse embryo brain at TS23 highlights the challenges that arise when dealing with morphological differences in the underlying models. To confront these issues, ontology-based and spatial rule-based mappings are also being explored (Figure 5d).

### The Whole Brain Catalog

**Figure 6:**
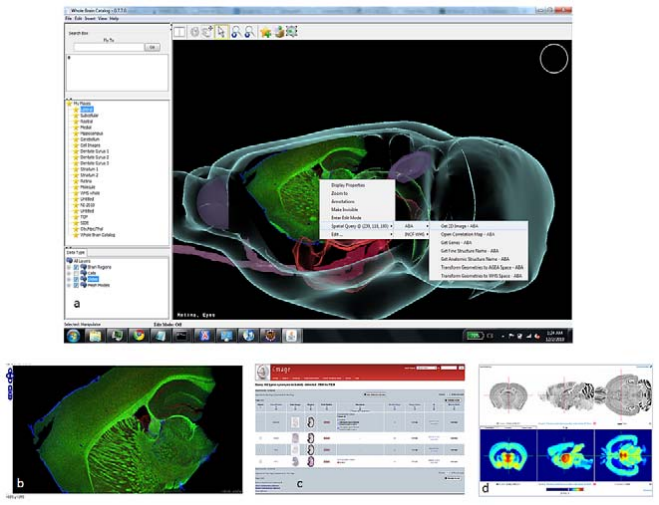
The Whole Brain Catalog. From the WBC 3-DAtlas Integration Client shown in (a), a user can generate a spatial query of WHS registered atlases. A probe can be placed in the 3-D space of the viewer and WHS coordinates of the probe translated into other atlas coordinate spaces. Implemented queries include (b) CCDB-UCSD, (c) EMAP/EMAGE, and (d) AGEA/ABA, enabling a framework for interchange between these atlases.

The WBC (http://wholebraincatalog.org/) is a multi-scale open source virtual catalog of the mouse brain and builds on core technologies from the NIH-Blueprint Neuroscience Information Framework and CCDB (http://ccdb.ucsd.edu/index.shtm) (Figure 6). WBC can employ WHS or the Allen Reference Atlas as one of its spatial reference frameworks and it accesses INCF-DAI Web services for the spatial localization of data across atlas hubs. The CCDB is a Web accessible database for high resolution 2-D, 3-D, and 4-D data from light and electron microscopy. Many of these high resolution images have been registered to a Web-based Paxinos and Franklin mouse atlas [1], [32] and can be queried via the Smart Atlas [35], [36] from within the WBC application. Through the WHS the WBC can now access ABA and EMAGE data as well. These resources were both developed at UCSD.

## The Future of Digital Atlasing

Constructing an open and shared digital atlasing framework has the potential to transform collaborative research. While building such a framework would be considerably more challenging in higher mammals, the benefits in the mouse, and rodent in general, are extraordinary and well worth the effort. A more mature system may be able to bridge the rich data sets from different research groups across different species, experimental modalities, and locations. As more groups tie their resources to this framework, it will be possible to access data and applications located at a researcher's spatial areas of interest. Infrastructure is being developed, so any client that includes known spatial information and uses INCF-DAI services can access these integrated resources.

Large-scale efforts are presently underway in the mouse for brain-wide experimental mapping of neural circuits at a mesoscopic resolution using injections of tracers or viral vectors [37], and via genetically modified lines. These connectional atlases will provide another level of understanding of brain architecture but will yield atlases of increasing complexity [38]. Dissemination and analysis of the data and created in these efforts may greatly benefit from implementing a standardized anatomic architecture such as proposed here. The INCF Digital Atlasing program is inspired by the vision described here, and plans to continue to create recommendations and standards that move the community toward this goal. Approaching standardization and infrastructure development for brain atlasing as a community effort will allow unprecedented data interchange and interoperability of resources that support our shared scientific goals.
