# Correction: Digital Atlasing and Standardization in the Mouse Brain

**DOI:** 10.1371/annotation/22c5808a-56cf-46e5-ba1b-456e838a5428

**Published:** 2011-02-11

**Authors:** Michael Hawrylycz, Richard A. Baldock, Albert Burger, Tsutomu Hashikawa, G. Allan Johnson, Maryann Martone, Lydia Ng, Chris Lau, Stephen D. Larsen, Jonathan Nissanov, Luis Puelles, Seth Ruffins, Fons Verbeek, Ilya Zaslavsky, Jyl Boline

The ninth author's name was spelled incorrectly. The correct name is: Stephen D. Larson.

